# COMPARING THE PREDICTIVE EFFICACY OF MELD AND ALBI SCORES IN LIVER CIRRHOSIS PATIENTS WITH ACUTE UPPER GASTROINTESTINAL BLEEDING

**DOI:** 10.1590/S0004-2803.24612024-075

**Published:** 2025-09-05

**Authors:** Zahra Shokati ESHKIKI, Razieh KHAZAEI, Abazar PARSI, Ali Akbar SHAYESTEH

**Affiliations:** 1 Alimentary Tract Research Center, Clinical Sciences Research Institute, Ahvaz Jundishapur University of Medical Sciences, Ahvaz, Iran.

**Keywords:** Liver cirrhosis, acute upper gastrointestinal bleeding, ALBI score, MELD score, mortality, Cirrose hepática, sangramento gastrointestinal alto agudo, escore ALBI, escore MELD, mortalidade

## Abstract

**Background:**

Acute upper gastrointestinal bleeding (AUGIB) is a critical medical emergency and is a common cause of illness and death in individuals with liver cirrhosis.

**Objective:**

The point of this study was to check how well the albumin-to-bilirubin ratio (ALBI) and model for end-stage liver disease (MELD) scores could predict how these patients would do in the future.

**Methods:**

The Imam Khomeini Hospital gastroenterology department conducted a retrospective examination. We admitted 102 patients with AUGIB and liver cirrhosis from April 2021 to September 2023. The study included a full medical history and clinical evaluation upon admission, as well as all laboratory test results throughout the hospital stay. We diagnosed liver cirrhosis using clinical, laboratory, and radiologic data. We diagnosed AUGIB as having hematemesis, melena, or hematochezia. We then tested the ALBI, MELD, and liver and kidney function. Some criteria allow continuous variable comparison, whereas others allow discrete variable comparison. Death during hospitalization and rebleeding were the key outcomes, with one-month mortality assessed. We compared ALBI and MELD before establishing their relationship to mortality and rebleeding.

**Results:**

Of the 102 patients, 68.5% survived. Upon arrival, we noted a markedly elevated prevalence of edema, ascites, and chilly extremities among patients who did not survive. The MELD and ALBI scoring systems effectively forecast in-hospital mortality. The threshold for MELD is 21 (CI: 0.759-0.930, P=0.00), whereas for ALBI it is -2.3 (CI: 0.865-0.950, P=0.01). Neither party could foresee hospitalization or premature rebleeding. The probability of death may be forecasted using the MELD during the first discharge phase (P<0.05).

**Conclusion:**

The MELD and ALBI scores show a suitable ability to predict short-term outcomes and both of them can predict death and rebleeding, as well as 1-month mortality. Nevertheless, we recommend that in individuals with advanced liver cirrhosis, the MELD score is a more accurate prognostic indicator compared to the ALBI score.

## HIGHLIGHTS

• This research sought to determine the predictive power of the albumin-to-bilirubin ratio (ALBI) and the model for end-stage liver disease (MELD) scores for these patients’ future performance.

• The ALBI and MELD scores demonstrate a reasonable capacity for short-term outcome prediction.

• The MELD and ALBI scores may predict both 1-month mortality and death and rebleeding.

• The findings suggest that the MELD score is a more reliable prognostic factor than the ALBI score in patients with advanced liver cirrhosis.

## RESUMO

**Contexto:**

A hemorragia digestiva alta aguda (HDAA) é uma emergência médica crítica e é uma causa comum de doença e morte em indivíduos com cirrose hepática.

**Objetivo:**

O objetivo deste estudo foi verificar o quão bem a relação albumina-bilirrubina (ALBI) e o modelo para doença hepática terminal (MELD) poderiam prever como esses pacientes se sairiam no futuro.

**Métodos:**

O departamento de gastroenterologia do Hospital Imam Khomeini realizou um exame retrospectivo. Admitimos 102 pacientes com AUGIB e cirrose hepática de abril de 2021 a setembro de 2023. O estudo incluiu um histórico médico completo e avaliação clínica na admissão, bem como todos os resultados de exames laboratoriais durante a internação. Diagnosticamos cirrose hepática usando dados clínicos, laboratoriais e radiológicos. Diagnosticamos AUGIB como tendo hematêmese, melena ou hematoquezia. Em seguida, testamos o ALBI, MELD e a função hepática e renal. Alguns critérios permitem a comparação contínua de variáveis, enquanto outros permitem a comparação discreta de variáveis. Morte durante a hospitalização e ressangramento foram os principais desfechos, com mortalidade avaliada em um mês. Comparamos ALBI e MELD antes de estabelecer sua relação com mortalidade e ressangramento.

**Resultados:**

Dos 102 pacientes, 68,5% sobreviveram. Na chegada, observamos uma prevalência acentuadamente elevada de edema, ascite e extremidades frias entre os pacientes que não sobreviveram. Os sistemas de pontuação MELD e ALBI preveem efetivamente a mortalidade intra-hospitalar. O limite para MELD é 21 (IC: 0,759-0,930, P=0,00), enquanto para ALBI é -2,3 (IC: 0,865-0,950, P=0,01). Nenhuma das partes poderia prever hospitalização ou ressangramento prematuro. A probabilidade de morte pode ser prevista usando o MELD durante a primeira fase de alta (P<0,05).

**Conclusão:**

Os escores MELD e ALBI mostram uma capacidade adequada de prever desfechos de curto prazo e ambos podem predizer morte e ressangramento, bem como mortalidade em 1 mês. No entanto, recomendamos que, em indivíduos com cirrose hepática avançada, o escore MELD seja um indicador prognóstico mais preciso em comparação com o escore ALBI.

## INTRODUCTION

Cirrhosis is the final stage of chronic liver disease, characterized by the development of severe symptoms such as portal hypertension (PH), ascites, jaundice, and encephalopathy^1^ ^,^ ^2^. In these patients, acute esophageal variceal bleeding (AEVB) is a significant cause of death due to PH. According to medical guidelines^3^ ^,^ ^4^, esophageal variceal band ligation (EVBL) is the standard treatment for AEVB. Despite achieving hemodynamic stabilization with blood transfusion and EVBL, there is a possibility of recurrent bleeding, which can ultimately result in the patient’s demise. Therefore, the severity of liver dysfunction and hemodynamic derangement determines the outcome and survival of patients with AEVB^5^ ^,^ ^6^.

Evaluating the prognosis of patients with liver cirrhosis is a crucial process, particularly in liver transplant clinics, as it helps prioritize patients on the waiting list. To achieve this objective, researchers have established multiple scoring systems, with the model for end-stage liver disease (MELD) and Child-Pugh being the most valuable instruments^7^ ^,^ ^8^. Researchers have developed the albumin-to-bilirubin ratio (ALBI) as a tool to predict the survival of patients with hepatocellular carcinoma (HCC) who also have cirrhosis. We use this tool in conjunction with the Barcelona classification^9^. Furthermore, it yielded satisfactory outcomes in evaluating liver damage following chemoembolization in patients with HCC^10^. Previous research has shown that this scoring system is better than MELD and Child-Pugh scores at predicting death from cirrhosis in people with primary biliary cholangitis (PBC) and hepatitis B virus (HBV) cirrhosis^11^ ^,^ ^12^. It did not perform as well as MELD in other situations, such as the post-transjugular intrahepatic portosystemic shunt (TIPS) operation^13^.

AEVB is a severe state characterized by a significant death rate and considerable financial burden. We commonly use the MELD and Child-Pugh grading systems to evaluate these individuals’ prognosis^14^. Few studies evaluated the ALBI score in acute upper gastrointestinal bleeding (AUGIB) and their results were similar to MELD and Child-Pugh in predicting the outcome of AEVB^15^. Therefore, we aimed to compare the prognostic performance and accuracy of ALBI and MELD scores in determining the survival and outcome of these critically liver cirrhotic patients.

## METHODS

### Study population

A retrospective investigation was conducted at AJUMS’ Imam Khomeini Hospital. The study included 102 patients admitted to the gastroenterology ward because of AUGIB and liver cirrhosis. Imam Khomeini Hospital in Ahvaz, Iran conducted this study, admitting patients from April 2021 to September 2023. Before their inclusion in the study, all patients provided informed consent, and the trial received approval from the Ethics Committee of Ahvaz Jundishapor University of Medical Sciences (Ethics Committee number: IR.AJUMS.REC.1394.122).

The research included a thorough medical history and clinical examination upon admission, as well as the results of all laboratory tests conducted throughout the hospital stay. The diagnosis of liver cirrhosis included the use of a mix of clinical, laboratory, and radiologic data. We also used hematemesis (vomiting blood or something that looks like coffee grounds), melena (black, tarry stools), or hematochezia (fresh blood going into the rectum) to diagnose AUGIB^16^ ^,^ ^17^. Next, we examined the liver and kidney function tests and complete blood count (CBC), assessed the MELD score, and measured the ALBI. Certain criteria permit the comparison of continuous variables, while others facilitate the comparison of discrete variables. Death during hospitalization and rebleeding were the main outcomes measured, with further assessment of mortality at 1 month. We assessed and compared the ALBI score and MELD, which we calculated using the standard formula, before determining their correlation with mortality and rebleeding.

### Data collection

Demographics (age, gender), hospital stays, and the etiology of liver disease (cryptogenic, hepatitis C virus (HCV), HBV, alcohol, and other reasons) were the main data gathered. Several lab tests were done, such as total bilirubin, albumin, liver transaminases (ALT, AST), alkaline phosphatase (ALK-P), platelet and leukocyte counts, total bilirubin, hemoglubin (Hb), hematocrit (HCT), creatinine (CR), albumin, prothrombin time (PT), activated partial thromboplastin time (aPTT), and international normalized ratio (INR). Finally, we gathered data on the existence and severity of esophageal varices. 

### Definitions and formulas

The MELD score is one of the prognostic systems which calculates according to the following equation: 

MELD score= 9.57 × loge (creatinine) + 3.78 × loge (total bilirubin) + 11.2 × loge (INR) + 6.43^18^.

ALBI score is also determined in patients living with cirrhosis according to the following standard formula:

ALBI score = (log10 bilirubin [µmol/L] × 0.66) + (albumin [g/L] × −0.0852). As a result, ALBI grades 1, 2, and 3 were developed as follows: ALBI score ≤ −2.60 (ALBI grade 1), > −2.60 to ≤ −1.39 (ALBI grade 2), and > −1.39 (ALBI grade 3)^19^.

We determined the MELD and ALBI scores based on the information we gathered about study participants upon referral. We calculated the areas under the receiver operating characteristic (ROC) curves for each of these scoring systems, along with their 95% confidence intervals (CI). Then, we used binary logistic regression to calculate the odds ratio (OR) and compare the accuracy of each scoring system. 

### Statistical analysis

We provided categorical data in terms of frequency (%) and reported continuous variables as medians (ranges). We used Fisher’s exact test, also known as the chi-square test, to compare these variables. We utilized Microsoft Windows SPSS for data analysis. We used the independent sample t-test, also known as the Mann-Whitney U test, to analyze quantitative data and determine statistical differences. We deemed P values significant if they were less than 0.05.

## RESULTS

All 102 patients living with cirrhosis with AUGIB who were admitted to the Gastroenterology Department of the Imam Khomeini Hospital, from April 2021 to September 2023, were enrolled in the study.

The mean age of the population was 63 years, 80 patients (78.4%) were male, the most common etiology was cryptogenic (37.3%) and the most common presenting symptom of AUGIB was hematemesis (38.3%).

According to Table 1, age, sex, etiology of End-stage liver disease (ESLD) and presenting symptoms were similar in survived and non-survived patients. (P>0.05). However, the presence of edema (P:0.047), ascites (P:0.003), and cold extremities (P:0.001) on admission time suggest more critical condition and a decrease in organ perfusion was significantly associated with higher mortality. 

**TABLE 1:** Patients’ characteristics.

Items	Liver cirrhosis	Survived patients	Non-survived patients	P value
Patients (n)	102	70	32	-
Age (y)	62.8 (25-88)			P>0.05
Male sex (%)				
Female sex (%)	78.4%			
21.6%			P>0.05	
Cause of cirrhosis				
Alcohol (%)	4.9%			P>0.05
Hepatitis B (%)	32.4%			P>0.05
Hepatitis C (%)				
Others or unknown (%)	22.5%			
40.2%				
33 (47.1%)				
8 (25%)	P>0.05			
P>0.05				
AUGIB’ symptom				
Hematemesis (%)	38.3%	46 (65.7%)	24 (75 %)	P>0.05
Melena (%)	30.4%	37 (52.8%)	23 (71.8%)	P>0.05
Hematochezia (%)	2.9%	1 (1.4 %)	2 (6.25%)	P>0.05
Clinical examination				
Edema	67.6%	43 (61.4%)	26 (81.2%)	0.047
Ascites	59.8%	35 (50%)	26 (81.3%)	0.003
Cold extremities	36.3%	18 (25.7%)	19 (59.3%)	0.001
Supine Systolic blood pressure	-	115.4±3.62 (60-180)	99.53±2.57 (60-160)	P>0.05
Supine heart rate	-	87.84±1.10 (48-140)	92.84±1.50 (56-126)	P>0.05

Data are the mean ± SEM (range) or the frequency of individuals (percentage).

However, supine systolic blood pressure (SBP) and heart rate (HR) weren’t associated with higher mortality.

Presence of hypertension (HTN), chronic kidney disease (CKD), coronary heart disease (CHD), peptic ulcer disease (PUD), previous AUGIB, and minimal alcohol drinks, using nonsteroidal anti-inflammatory drug (NSAID) and Aspirin didn’t affect the survival of patients (Table 2). However, smoking had a serious effect and significantly decreased patient survival (P:0.02).

**TABLE 2:** Comorbidities/risk factors and patient’s mortality.

Records	Survived patients (n=70)	Non survived patients (n=32)	P value
Diabetes	22 (31.4%)	7 (21.9%)	P>0.05
Hypertension	18 (25.7%)	8 (25%)	P>0.05
CKD	4 (5.7%)	1 (3.1%)	P>0.05
AUGIB	55 (78.6%)	29 (90.6%)	P>0.05
CHD	1 (1.4%)	1 (3.1%)	P>0.05
PUD	2 (2.9%)	1 (3.1%)	P>0.05
Cigarette smoking	31(44.3%)	22 (68.8%)	P>0.05
Alcohol	7 (10%)	3 (9.4%)	P>0.05
Drug-using history			
ASA	5 (7.1%)	3 (9.4%)	P>0.05
NSAID	5 (7.1%)	4 (12.5%)	P>0.05

Data are the mean or the frequency of individuals (percentage). CKD: chronic kidney disease; AUGIB: acute upper gastrointestinal bleeding; CHD: coronary heart disease; PUD: peptic ulcer disease; ASA: acetylsalicylic acid; NSAID: nonsteroidal anti-inflammatory drug.

Patients who took NSAIDs had more in-hospital rebleeding than those who didn’t use it (P:0.01). 

Patients who didn’t survive had more intensive care unit (ICU) admissions, blood product transfusions and more units of blood transfusions (P<0.05). Non-survived patients had significantly more in-hospital rebleeding (P:0.02), higher MELD score (P<0.01) and higher ALBI (P<0.01) (Table 3).

**TABLE 3:** Patients’ clinical features and prognosis.

Clinical features	Survived patients (n=70)	Non-survived patients (n=32)	P value
ICU admission time	0 (0-7)	3 (0-13)	P<0.01
Needing to receive blood	43 (61.4%)	27 (84.4%)	P>0.01
Transfused blood Units	2 (0-10)	3 (0-10)	P<0.01
Re-bleeding during admission	10 (14.2%)	11 (34.4%)	P>0.01
MELD score	16 (7.00-38.00)	29 (10.00-39.00)	P<0.01
ALBI score*	-3.1±0.68	-2.5±0.07	P<0.01

Data are the median (range) or the frequency of individuals (percentage). *Data are the mean ± SEM. ICU: intensive care unit.

Both the MELD score and ALBI couldn’t predict the risk of rebleeding during hospital admission. Table 4 (P>0.05). 

**TABLE 4:** Patients’ prognostic values and rebleeding.

Clinical features	Patients with re-bleeding (n=21)	Patients without re-bleeding (n=81)	P value
MELD score	22±0.65	18±2.40	P>0.05
ALBI score	-2.6±0.08	-2.9±0.25	P>0.05

Data are the mean ± SEM.

Esophagogastroduodenoscopy (EGD) was done in 101 patients which revealed that esophageal varices were the most common finding and then followed by Erosions and Peptic ulcers. High-grade varices were more common than low-grade varices in both groups and did not have a significant difference between survived and non-survived groups. (Table 5), All of the patients in the non-survived group had Esophageal varices and all of the non-variceal bleedings were alive at the end of admission.

**TABLE 5:** Patients’ endoscopy information and mortality rate.

Endoscopy information	Survived patients (n=70)	Non-survived patients (n=32)	P value
Endoscopy findings			
Ulcer	6 (100%)	0 (0.0%)	P <0.05
Erosion	7 (100%)	0 (0.0%)	P <0.05
Varices	57 (67.1%)	31 (32.9%)	P <0.05
Variceal Grade			
High grade	51 (66.7%)	26 (33.3%)	P >0.05
Low grade	6 (60%)	5 (40%)	P >0.05
Therapy			
Band ligation	55 (78.5%)	27 (84%)	P >0.05
APC	4 (100%)	0 (0.00%)	P >0.05
PPI	10 (76.9%)	3 (23.1%)	P >0.05
Black more tube	1 (33.3%)	2 (66.7%)	P >0.05

Data are the median (range) or the frequency of individuals (percentage). APC: argon plasma coagulation; PPI: proton pump inhibitor.

The correlation between the laboratory findings and in-hospital mortality revealed that higher levels of INR, PT, PTT, bilirubin, creatinine and BUN were significantly associated with higher rates of death. On the other hand, lower levels of albumin (ALB), hematocrit (HCT) and hemoglobin (Hb) significantly increased in-hospital death. (Table 6, P<0.05).

**TABLE 6:** Laboratory characteristics of patients.

Laboratory data	Survived patients (n=70)	Non-survived patients (n=32)	P value
Hb (g/dL)	9.00±0.95 (4.6-14.2)	8.08±0.57 (4.3-13.8)	P>0.01
HCT (%)	26.97±1.83 (14.50-42.00)	23.90±1.49 (14.20-39.00)	P≥0.01
BUN (mg/dL)	32.98±2.59 (11.00-121.00)	40.53±4.78 (11.00-140.00)	P>0.01
Creatinine (mg/dL)	1.4±0.06 (0.7-5.5)	1.95±0.09 (0.7-4.4)	P<0.01
Bilirubin (mg/dL)	2.78±0.95 (0.5-8.60)	5.10±0.81(1.16-6.32)	P<0.01
Albumin (g/dL)	2.99±0.09 (2.00-4.20)	2.49±0.07 (1.70-4.10)	P<0.01
PT (s)	15.87±1.14 (12.00-35.00)	21.08±1.25 (12.00-34.00)	P<0.01
INR	1.71±0.05 (1.00-6.70)	2.76±0.24 (1.16-6.32)	P<0.01
PTT (s)	41.08±2.87 (23.60-120.00)	65.89±3.33 (28.00-120.00)	P<0.01

Data are the mean ± SEM. Hb: hemoglobin; HCT: hematocrit; BUN: blood urea nitrogen; PT: prothrombin time; INR: International Normalization Ratio; PTT: partial thromboplastin time.

Seventy patients discharged and followed for 1 month, 36 (51.5%) had re bleeding in this period. Risk of Re-bleeding didn’t increase by the presence of ascites, high grade varices and higher MELD score (Table 7, P>0.05). 

**TABLE 7:** Independent variables and re-bleeding after follow up.

Variables	Patients with re-bleeding (n=36)	Patients without re-bleeding (n=34)	P value
Ascites	21 (60%)	14 (40%)	P>0.05
Varices	31 (54.4%)	26 (45.6%)	P<0.05
High grade varices	23 (53.3%)	19 (46.7%)	P>0.05
MELD score*	18±0.76	15±1.6	P>0.05

Data are the median (range) or the frequency of individuals (percentage). *Data is the mean ± SEM.

Independent variables also affected the survival of the patients with the following features: male gender, hypertension, and higher levels of blood urea nitrogen (BUN), creatinine, prothrombin time (PT), International Normalized Ratio (INR), and MELD scores were associated with high mortality (Table 8).

**TABLE 8:** Independent variables and mortality after 1 month follow-up.

Variables	Survived patients (n=61)	Non-survived patients (n=9)	P value
Male gender	49 (80%)	4 (44.4%)	P<0.05
Hypertension	13 (21.3%)	5 (55%)	P<0.05
BUN (mg/dL)	29.24±16.26 (11-75)	58.33±39.87 (15-11)	P<0.05
Creatinine (mg/dL)	1.27±0.50 (0.70-3.10)	2.30±1.58 (1.00-5.50)	P<0.05
Albumin (g/dL)	2.98±0.58 (2.00-4.20)	3.03±0.64 (2.00-4.10)	P>0.05
PT (s)	15.25±3.33 (12-33)	19.37±6.91 (13.10-35.00)	P<0.05
INR	1.59±0.78 (1.00-6.70)	2.46±1.55 (1.10-6.00)	P<0.05
High-grade varice	35 (83.3%)	7 (16.7%)	P>0.05
MELD score*	15 (7-34)	27 (10-38)	P<0.05

Data are expressed as the mean ± SD. *Data is the median. BUN: blood urea nitrogen; PT: prothrombin time; INR: International normalized ratio.

**FIGURE 1:**
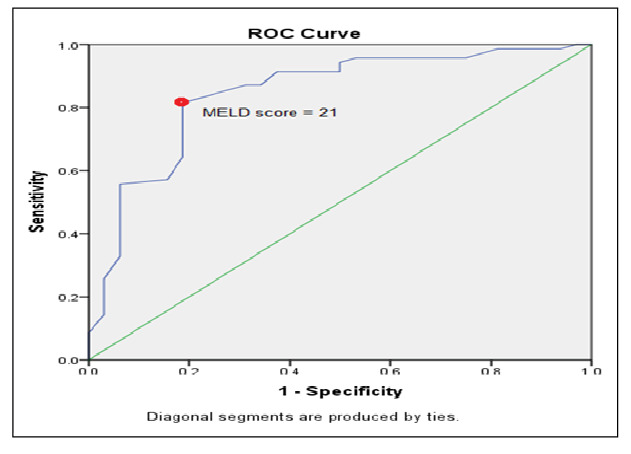
ROC analysis of MELD scores for predicting the in-hospital mortality in AUGIB.

MELD was assessed as a mortality predictor in patients living with cirrhosis with AUGIB. Concerning the in-hospital overall mortality rate, the cutoff value of the MELD score was 21 (CI 0.759-0.930, P=0.00) with a specificity of 81.2% and a sensitivity of 81.4% (Figure 1).

AUGIB: Acute upper gastrointestinal bleeding.

**FIGURE 2:**
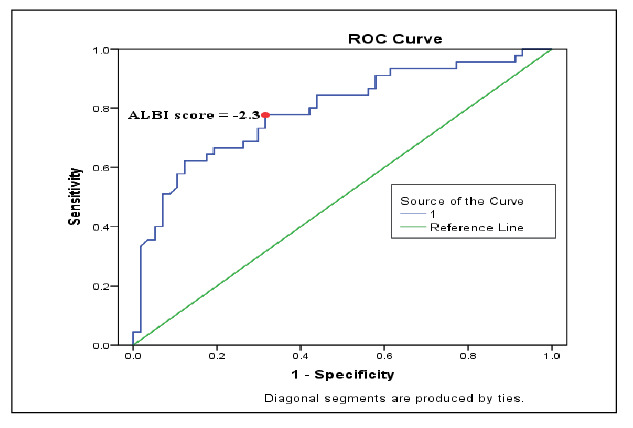
ROC analysis of ALBI scores for predicting the in-hospital mortality in AUGIB.

ALBI: albumin-to-bilirubin ratio; AUGIB: Acute upper gastrointestinal bleeding.

ALBI score was also assessed as a mortality predictor. The cutoff value of the ALBI score was 2.3 (CI 0.865-0.950, P=0.01) with a specificity of 82.1% and a sensitivity of 79.8% (Figure 2).

**FIGURE 3:**
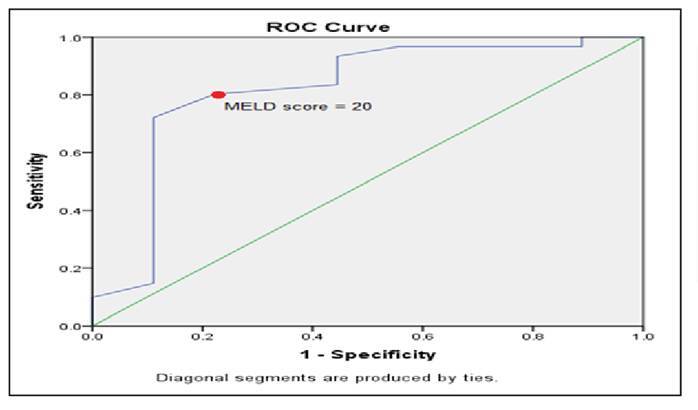
ROC analysis of MELD scores for predicting the mortality in discharged patients.

After 1 month follow up the MELD scores of discharged patients were as follows: Cut off the value of 20 (CI: 0.643-0.996, P=0.002), a specificity of 77.8% and a sensitivity of 80.2% (Figure 3). The analysis was conducted for the ALBI scores of discharged patients; however, after one month of follow-up, the ALBI scores of discharged patients did not show statistical significance. 

Binary logistic regression also was used to assess for predictors of the outcomes. MELD and ALBI scores as prognostic systems were independently associated with the mortality rate (like other independent variables in Table 9).

**TABLE 9:** Independent variables and in-hospital mortality in AUGIB.

Variables	Crude OR	Confidence interval	P value
Ascites	4.33	1.58-11.82	P<0.01
Albumin	0.23	0.09-0.52	P<0.01
MELD score	1.20	1.12-1.29	P<0.01
ALBI score	0.17	0.07-0.39	P<0.01

AUGIB: Acute upper gastrointestinal bleeding.

## DISCUSSION

While many studies^15^ ^,^ ^20^ have looked at AEVB in-hospital mortality rates in people with cirrhosis, not many have looked at how useful the ALBI and MELD scores are for predicting these rates. We discovered that, in contrast to the current research, the MELD score and the ALBI score had similar predictive efficacy for predicting in-hospital mortality in patients with ESLD^20^. Significantly, our findings indicate that the MELD score serves as a more precise predictive indicator for assessing short-term outcomes in these individuals than the ALBI score. 

A prominent complication that modifies the natural course of both compensated and decompensated cirrhosis^21^ is the onset of an episode of AUGIB, which is also a major source of morbidity and death in these patients. Our research, like other studies, found that patients had a higher risk of dying if they had a lot of problems with their liver if they were bleeding or not if the bleeding was severe, if they had HCC or hepatic encephalopathy, or if they had other health problems^22^. The results of this investigation showed that although ALBI was marginally more accurate in predicting hospital deaths, neither model was able to predict rebleeding events. In their investigations, Zou D et al. found that although ALBI’s AUC was greater than the others, MELD, Child-Pugh, and ALBI were all equally effective in predicting mortality^23^. However, in their research on alcoholic cirrhosis patients, Nagaraja BS et al. discovered that MELD and Child-Pugh were better than ALBI in predicting the fate of AEVB patients^24^. Although our study’s cutoffs were lower, the AUC for MELD and ALBI was greater.

Furthermore, Ellsharawy O et al. in Egypt demonstrated that MELD was less accurate than ALBI in predicting both short-term mortality and rebleeding^25^. In our study, however, the presence of edema, ascites, and cold extremities during the examination upon hospital arrival were strong predictors of patient mortality; these were indicators of the severity of bleeding or chronic liver disease. The effect of age (63 years old) and sex (78% male) on mortality is defined in previous studies as well as in our study. We have not applied these findings in MELD or ALBI, and they are associated with the severity of bleeding. Furthermore, there was a greater death rate in these individuals who smoked (>0.05). While acetylsalicylic acid (ASA) users fared better than non-users in terms of survival, other NSAID users had far higher death rates, consistent with earlier research^26^ ^-^ ^28^.

The second-most notable discovery was the association between hospital course (length of ICU stay, need for blood or blood product transfusion, in-hospital rebleeding, MELD and ALBI scores upon arrival) and death rate in AUGIB in cirrhosis patients. Furthermore, AUGIB, as an independent risk factor, predisposes individuals to additional cirrhosis problems, such as hepatorenal syndrome, a condition closely associated with a poor short-term prognosis^29^. Moreover, significant bleeding may result in hypovolemic shock and high blood transfusion needs which can lengthen ICU stays, increase rebleeding episodes, and worsen patients’ conditions as measured by MELD and ALBI scores^30^.

We found that disorders other than esophageal varices caused 14% of AUGIB in cirrhosis patients, and all of them recovered. Thus, similar to the del Olmo JA et al. research, the lack of EV on endoscopy was a positive prognostic indicator in our patients^31^. 

We found a link between a higher risk of AUGIBs dying and changes to laboratory tests that indicate liver dysfunction, including INR, PT, PTT, bilirubin, creatinine, and BUN. The MELD and ALBI scores^32^ ^,^ ^33^ have the potential to predict hospital mortality because they indicate the degree of cirrhosis^15^ ^,^ ^34^. High-grade varices, MELD score, INR, PT, creatinine, BUN, and albumin were all associated with one-month survival. As a result, individuals with cirrhosis who have higher MELD and ALBI scores should expect a significant increase in AUGIB severity, morbidity, and mortality.

Additionally, we found an independent link between the patient’s outcome after AUGIBs and the prognostic scheme of MELD and ALBI scores, along with other independent factors like albumin and ascites. The patients’ AUGIB-related mortality predictors were comparable to those of non-AUGIB patients. These indicators measure survival both during hospitalization and four weeks after release. Compared to previous studies, our patients’ lower liver function reserves and overall health status contribute to a higher death rate. In our investigation, the patients’ MELD and Child-Pugh scores were significantly higher. For MELD and ALBI, the cutoff values for hospital mortality were 22 and -2.3, respectively. Additionally, patients with milder illnesses at admission are also at risk after release, as shown by the cutoff value for the MELD score, which was 21 for the 4-week period. The research conducted by Zuli D et al. revealed that the MELD and ALBI scores had considerably lower cut-off values (9.5 and −1.5237 for MELD and ALBI, respectively), which might perhaps be attributed to the study population^35^ ^-^ ^38^. 

Interestingly, our data also demonstrated that, in terms of short-term results, the ALBI scoring method was almost identical to the MELD score. 

## CONCLUSION

The key outcomes measured in this trial were mortality during hospitalization and rebleeding, as well as mortality after one month in patients with AUGIB and liver cirrhosis. We evaluated and contrasted the ALBI score and MELD before establishing their link with mortality and rebleeding. This research indicates that the ALBI score is as practical and effective as the MELD score for evaluating short-term results. The MELD scoring system shows how higher levels of creatinine and INR affect people with severe liver diseases. This finding suggests that this model may be better at predicting death in people with advanced liver cirrhosis than the ALBI score. Nonetheless, further investigation in this domain is necessary to substantiate these conclusions. 
